# Diffraction data of core-shell nanoparticles from an X-ray free electron laser

**DOI:** 10.1038/sdata.2017.48

**Published:** 2017-04-11

**Authors:** Xuanxuan Li, Chun-Ya Chiu, Hsiang-Ju Wang, Stephan Kassemeyer, Sabine Botha, Robert L. Shoeman, Robert M. Lawrence, Christopher Kupitz, Richard Kirian, Daniel James, Dingjie Wang, Garrett Nelson, Marc Messerschmidt, Sébastien Boutet, Garth J. Williams, Elisabeth Hartmann, Aliakbar Jafarpour, Lutz M. Foucar, Anton Barty, Henry Chapman, Mengning Liang, Andreas Menzel, Fenglin Wang, Shibom Basu, Raimund Fromme, R. Bruce Doak, Petra Fromme, Uwe Weierstall, Michael H. Huang, John C. H. Spence, Ilme Schlichting, Brenda G. Hogue, Haiguang Liu

**Affiliations:** 1 Complex Systems Division, Beijing Computational Science Research Center, ZPark II, Haidian, Beijing 100193, China; 2 Department of Engineering Physics, Tsinghua University, Beijing 100086, China; 3 Department of Chemistry, National Tsing Hua University, Hsinchu 30013, Taiwan; 4 Max-Planck-Institut für Medizinische Forschung, Jahnstraße 29, 69120 Heidelberg, Germany; 5 Biodesign Center for Immunotherapy, Vaccines and Virotherapy, Arizona State University, Tempe, Arizona 85287, USA; 6 Biodesign Center for Applied Structural Discovery, Arizona State University, Tempe, Arizona 85287, USA; 7 School of Molecular Sciences, Arizona State University, Tempe, Arizona 85287, USA; 8 Department of Physics, Arizona State University, Tempe, Arizona 85297, USA; 9 Linac Coherent Light Source (LCLS), SLAC National Accelerator Laboratory, Menlo Park, California 94025, USA; 10 Center for Free Electron Laser Science, Deutsches Elektronen-Synchrotron DESY, 22607 Hamburg, Germany; 11 Swiss Light Source, Paul Scherrer Institute, CH-5232 Villigen, Switzerland; 12 School of Life Sciences, Arizona State University, Tempe, Arizona 85287, USA

**Keywords:** Nanoparticles, X-ray crystallography

## Abstract

X-ray free-electron lasers provide novel opportunities to conduct single particle analysis on nanoscale particles. Coherent diffractive imaging experiments were performed at the Linac Coherent Light Source (LCLS), SLAC National Laboratory, exposing single inorganic core-shell nanoparticles to femtosecond hard-X-ray pulses. Each facetted nanoparticle consisted of a crystalline gold core and a differently shaped palladium shell. Scattered intensities were observed up to about 7 nm resolution. Analysis of the scattering patterns revealed the size distribution of the samples, which is consistent with that obtained from direct real-space imaging by electron microscopy. Scattering patterns resulting from single particles were selected and compiled into a dataset which can be valuable for algorithm developments in single particle scattering research.

## Background & Summary

The determination of structures of non-crystalline single molecules or nanoparticles from coherent diffractive imaging data is an exciting and rapidly developing field at X-ray Free-Electron Lasers (XFELs)^1^. Theoretical simulations^2^ predict that high resolution structure determination is feasible, but so far only two relatively low resolution three-dimensional (3D) reconstructions using single particle scattering method have been reported^3,4^. To advance and speed up XFEL-based single particle structure determination, an international collaboration (the single particle imaging initiative or SPI) was organized under the leadership of LCLS^5^. High resolution measurements of rice dwarf virus were reported recently^6^ but no 3D structure has been obtained yet for this virus. The 3D model reconstruction requires particle orientation determination before data averaging to improve the signal to noise ratio at high scattering angles, and further merging into a finely sampled 3D-diffraction volume. This is particularly challenging for the weakly scattering biological particles. We therefore set out to image core-shell heterostructures with both polyhedral cores and shells. These metal nanoparticles were chosen as a model system to mimic viruses containing differently shaped outer (capsid) and inner (genome) structures. The different symmetries of the outer shell and inner core are clearly reflected in the diffraction patterns. They can therefore serve as a test bed for algorithm development.

Synthetic metal nanoparticles have been used previously as model systems for developing imaging technology^3,7–12^. They have three major advantages: (1) the high-Z atoms have large scattering factors, resulting in stronger signals at higher resolution than same-sized biological particles; (2) metal nanoparticles can be prepared by a crystalline growth approach that frequently generates surface facets that lead to distinct streaks to assist merging; (3) nanoparticles can be manufactured with high symmetry. Together, these properties facilitate model reconstructions and data merging from a small number of patterns. As in biological systems, a potential problem with synthetic nanoparticles is the heterogeneity due to size variations and different compositions in the core-shells. This complicates orientation assignment and merging of diffraction patterns from differently-sized and ‘filled’ particles. Algorithms to disentangle orientation and particle composition/conformation are therefore needed to sort out the correct orientation for any particle.

**Figure 1:**
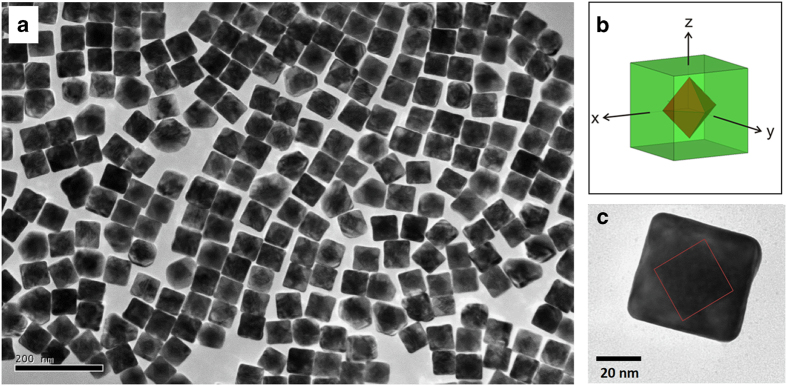
Core-shell nanoparticle sample information. (**a**) Area of sample particles under scanning/transmission electron microscope; (**b**) Schematic drawing of Au-Pd core-shell nanoparticle. The green cube indicates the outer shell of the particle composed of palladium, and the red coloured core is composed of gold. (**c**) A zoom in view of single particle viewed by electron microscopy. The red box depicts the gold core boundary.

Recently, protocols to synthesize homogeneously sized and shaped nanoscale core-shell particles have been established^13^. Here, we used Au-Pd core-shell particle (Fig. 1) as a model system to study XFEL scattering patterns. Using bright XFEL pulses, we collected 54,405 scattering patterns after preliminary data screening. Subsequently, based on the angles formed by two crossing series of fringes in the scattering patterns, the nanoparticle orientations with one facet perpendicular to the incident X-ray beam can be distinguished from other orientations. From the spacing between speckles of the interference fringes (the particle ‘shape-transform’), the size of the particles can be calculated. This allows us to compare the results with the size distribution obtained by scanning and transmission electron microscopy imaging. The Bragg spots from these nanocrystals occur at higher scattering angles that are not recorded by the detector with the geometry used for this reported measurement, so they could not be used in the analysis for data merging.

The data were compiled in the CXIDB format^14^ and deposited for public usage. This dataset should serve as a valuable asset for the development of algorithms in scattering pattern classifications, sample size analysis, 3D diffraction volume merging, and model reconstructions.

## Methods

### Sample preparation

Au-Pd core-shell nanocrystals were prepared at National Tsinghua University as previously described in detail by Yang *et al.*^13^. The particles were prepared in two stages. Briefly, Au octahedron shaped nanocrystals were first synthesized by mixing NaBH_4_ and HAuCl_4_ solutions to form gold seed particles. Solutions of cetyltrimethylammonium chloride (CTAC), HAuCl_4_ and KI were added to grow the gold particles to octahedral particles of desired sizes. The gold octahedron solution was then mixed with PdCl_2_, HCl and cetyltrimethylammonium bromide (CTAB) in deionized water. The mixture was stirred for about 12 h after adding ascorbic acid solution to allow the Au-Pd core-shell particles to form. The particles are composed of a cubic palladium shell, enclosing a regular octahedral gold core. The palladium shell exhibited a mean length of 52 nm (maximum 58 nm, minimum 48 nm), based on which, the mean edge length of gold cores was about 37 nm (Fig. 1).

### Sample injection and data collection

The experiments were performed in the nanofocus chamber of the Coherent X-ray Imaging (CXI)^15^ end station at the LCLS during Run 7, experiment L711. A gas dynamic focusing virtual nozzle injector^16^ (GDVN) was used to deliver the Au-Pd nanoparticles in few-micrometer wide liquid jet into the LCLS beam. An anti-settling system consisting of a slowly rotating temperature-controlled syringe holder was used to avoid particle sedimentation^17^. The photon energy was 6 keV (wavelength 2.06 Å), and the electron bunch duration was about 60 femtoseconds, generating XFEL pulses with shorter durations.

A Cornell-SLAC Pixel Array Detector (CSPAD) detector was positioned at 565 mm downstream of the XFEL-sample interaction point. The CSPAD detector panels were arranged in a geometry covering effectively an area of 1,748×1,748 pixels, with a pixel size of 110 μm×110 μm. The important parameters for the experiment are summarized in Table 1.

### Data analysis

**Figure 2:**
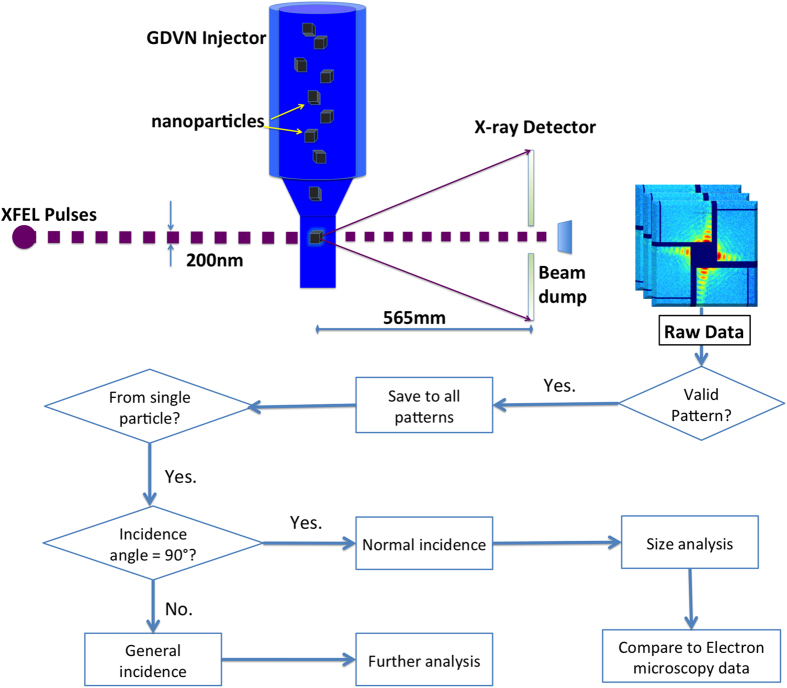
Scheme of the experimental setup and data analysis flow.

The data analysis flow is summarized in Fig. 2. The raw data were screened to select valid scattering patterns (‘hit-finding’), which were subsequently analysed to select the patterns that correspond to single particles. A subset of patterns with strong scattering streaks, which resulted from facets of cubic shell, was processed to obtain particle size information.

#### Hit-finding

The raw data is in the LCLS native XTC format. To identify the patterns with scattering signals resulting from nanoparticles, the programs CASS^18^ and Cheetah^19^ were used for on-line analysis, including preliminary hit-finding and hit-rate monitoring, in order to help optimize experimental parameters. Cheetah was used for post-experiment data analysis to select the scattering patterns of nanoparticles intercepted by XFEL pulses, i.e., the ‘hits’. The criterion used for identifying hits was the number (N_p_) of pixels with values that exceed the given threshold (I_th_). In this study, the threshold value, I_th_, was set to be 500 ADU (analogue to digital units). Patterns with N_p_ larger than 500 were classified as nanoparticle scattering patterns.

#### Defining the region of interest

In the low-resolution region, the gap in the center of detector resulted in missing low angle data. In the high-resolution region, signal from sample particles could not be distinguished from the background scattering. In a recently published work on rice dwarf virus particle scattering experiment, the samples were delivered to the chamber using aerosol injector, the background scattering is reduced to minimum, so the high resolution signals can be identified^5^. For the core-shell nanoparticle cases, the region with strong signal was effectively an annulus bounded by two circles with radii of 75 and 150 pixels, corresponding to 14.11 to 7.05 nm in terms of real-space resolution (d=λ/Θ, where Θ is the total scattering angle in the small-angle approximation, and where the momentum transfer q=2 π/d). The analysis presented in this work focuses to this region. The dataset for the region of interest covers a detector area of 401×401 pixels around the scattering center.

#### X-ray incidence angle calculation and size analysis

The facets of the outer cubic shell resulting in distinct scattering signatures that can be identified as series of fringes, forming streaks with strong signals. Note that the scattering from the waterjet also results in bright streaks, which should be masked out for data analysis. The difference is that the streaks due to nanoparticle surfaces are composed of recognizable fringes. If two series of fringes are perpendicular to each other, the incidence X-ray beam is normal to one of the cube facets. The spacing between fringes can be converted directly to the length of the particles in such ‘normal incidence’ cases. Based on the experimental geometry, the particle size (edge length, d) can then be calculated as,, where λ is the X-ray wavelength, *R* is the distance from the sample to the detector and Δx is the spacing between adjacent fringes on the detector^8^. For the cases where the incidence angle is not perpendicular to the cube surface, the procedure for the determination of orientation and the calculation of particle size are more complicated. More extensive analysis and results will be reported elsewhere.

#### Comparison with simulation data

A particle consisting of an octahedral core and a cubic shell was constructed as a reference model. The simulated scattering intensity was calculated using Fourier transforms in the projection approximation, neglecting Ewald sphere curvature (valid in the region of interest defined above). Simulated patterns were generated by slicing through Fourier space at desired orientations. The patterns were scaled using the particle size information to match the experimental speckles (the q-spacing between fringes).

### Code availability

The programs for hit-finding are publicly available^18,19^. The program for pattern classifications and size analysis was developed using Python, and can be downloaded from http://liulab.csrc.ac.cn/dokuwiki/doku.php?id=software.

## Data Records

**Figure 3:**
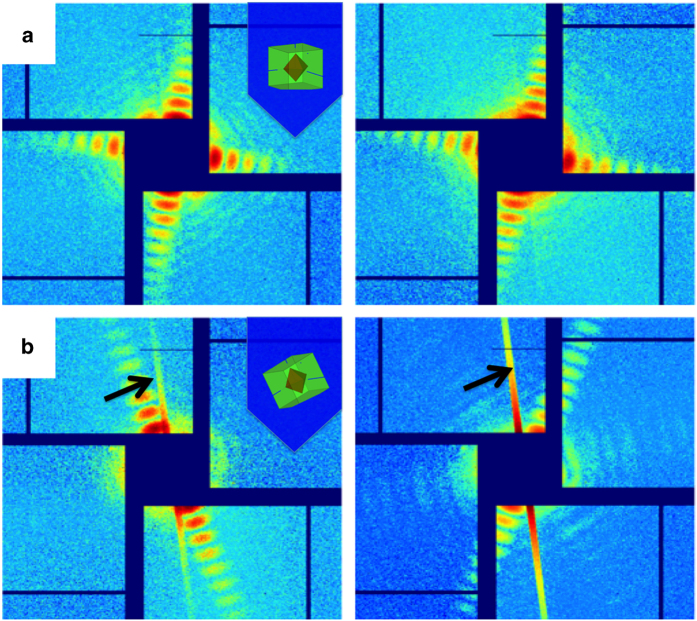
Representative scattering patterns. (**a**) Single particle scattering patterns at normal incidence angles; (**b**) Single particle scattering patterns at tilted incidence angles (general incidence cases). The inlet schematic drawing shows the orientation of particle with respect to the incident X-ray direction. Note that the arrows in (**b**) indicate the waterjet scattering signals, which must be masked out before data analysis.

There are two sets of data deposited to the CXIDB website, with the CXIDB ID 57, at http://cxidb.org/id-57.html (Data Citation 1). The first dataset contains all valid patterns (54,405) found in the primary screening process. The raw data from the CSPAD detector are converted to CXIDB format^14^. The directories are named by following LCLS experimental conventions, run numbers are indicated in the directory name; and the actual patterns identified by Cheetah are saved in the subdirectories, named ‘data1’, ‘data2’, etc., up to 1,000 patterns are saved in each data directory. The second dataset contains the subset of the patterns with fringe features reflecting from the cube facets. This dataset contains a representative subset of patterns exhibiting features of core-shell particles. The subset data, the region of interest that covers the central 401×401 pixels is stored using the format of CXIDB (Fig. 3). The auxiliary files include a README document and geometry description file in the CrystFEL format. Note that in spite of different extension names (h5 for the full dataset, cxi for the subset), the data are stored following HDF5 standard.

## Technical Validation

The scattering patterns of single particles are classified into ‘normal incidence’ and ‘general incidence’ cases, based on the angle between the incident X-ray beam direction and the surface of the cubic shell (Fig. 3). The ‘normal incidence’ cases can be directly subjected to particle size analysis. The ‘general incidence’ cases have to be processed using more complicated algorithms. It is worth noting that the continuous liquid jet caused background scattering, which is observable as strong needle-shaped streaks in Fig. 3. Correction of this background scattering has to be carried out before further analysis can be done. In this study, the water scattering was masked out prior to the pattern classification analysis.

**Figure 4:**
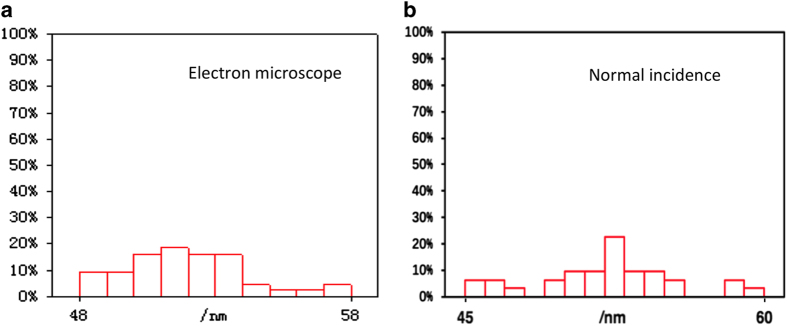
Particle size distributions. Size distribution obtained by scanning electron microscopy (**a**) versus that from XFEL scattering patterns at normal incidence angles (**b**).

The Au-Pd core-shell particles were examined by scanning and transmission electron microscopy. The cubic shell and octahedral core were visualized and the particle size distribution information was obtained from the electron microscopy images. For the XFEL scattering patterns, using the relation between particle size and the q-spacing between speckles in scattering patterns, the particle size distribution was calculated. The results from scanning transmission electron microscopy micrographs and the XFEL scattering patterns are consistent, as shown in Fig. 4. The average sizes of the particles obtained from STEM and XFEL data are very close. The size distribution obtained from STEM has an average value of 52 nm, ranging from 48 to 58 nm. Based on the particle size calculated from 209 scattering patterns that were classified as ‘normal incidence’ cases, the results revealed a size range from 45 to 60 nm, with a mean value of 52 nm.

**Figure 5:**
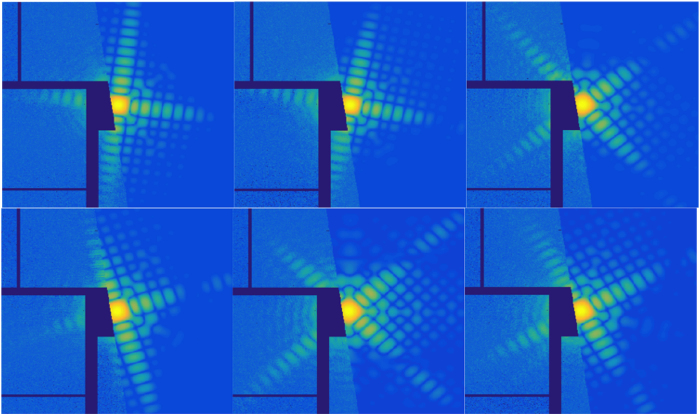
Comparison of experimental patterns with simulation data. The left halves of each pattern are from experimental data and the right halves are from simulations.

If the orientations and the sizes of core-shell nanoparticles can be recovered from the patterns, then the simulated patterns can be directly compared with experimental data. For the case of normal incidence X-rays, six representative examples where the experimental patterns nicely match the simulation data of the core-shell particles are shown in Fig. 5.

**Figure 6:**
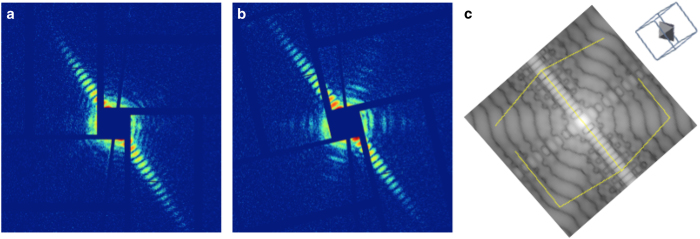
Features in scattering patterns of core-shell nanoparticles. (**a**) scattering pattern with no visible feature of Au core. (**b**) scattering pattern with visible feature of Au core. (**c**) simulated pattern from a core-shell particle at an orientation that matches the experimental case shown in (**b**), indicated in the inlet.

It is worthwhile to note that the nanoparticles not only differ in size, but also vary in terms of composition. The fraction of a given particle that is taken up by the Au-core can vary from particle to particle. In the extreme case, the particles may have very small cores, though the outer shells have similar sizes. Figure 6 shows two representative patterns for particles at similar orientations but with different features of the cores scattering signals, which can be compared to the simulated pattern at the same orientation (Fig. 6c).

## Usage Notes

The scattering patterns that passed our initial screening, i.e., the ‘hit-finding’, are stored in HDF5 format, following the convention of CXIDB. Patterns with clear facetted features of the core-shell particles were selected and saved in a separate file in CXIDB format. The standard API library for HDF5 is publicly available.

The orientations of the particles must be recovered to merge the single scattering patterns into 3D reciprocal space. Since the particles were delivered with a continuous liquid jet, the solvent scattering should be considered when a more in-depth interpretation of the data is carried out.

## Additional information

**How to cite this article**: Li, X. *et al.* Diffraction data of core-shell nanoparticles from an X-ray free electron laser. *Sci. Data* 4:170048 doi: 10.1038/sdata.2017.48 (2017).

**Publisher**’**s note**: Springer Nature remains neutral with regard to jurisdictional claims in published maps and institutional affiliations.

## Supplementary Material



## Figures and Tables

**Table 1:** Parameters for the Experiments.

**Quantity**	**Value**
X-ray Wavelength	2.06 Å
XFEL pulse duration	≤60 fs
X-ray focus (diameter of cross section)	~100 nm
Sample to detector distance	565 mm
Detector pixel size	110 μm
