# Corrigendum: Diffraction data of core-shell nanoparticles from an X-ray free electron laser

**DOI:** 10.1038/sdata.2017.154

**Published:** 2017-10-24

**Authors:** Xuanxuan Li, Chun-Ya Chiu, Hsiang-Ju Wang, Stephan Kassemeyer, Sabine Botha, Robert L Shoeman, Robert M Lawrence, Christopher Kupitz, Richard Kirian, Daniel James, Dingjie Wang, Garrett Nelson, Marc Messerschmidt, Sébastien Boutet, Garth J Williams, Elisabeth Hartmann, Aliakbar Jafarpour, Lutz M Foucar, Anton Barty, Henry Chapman, Mengning Liang, Andreas Menzel, Fenglin Wang, Shibom Basu, Raimund Fromme, R.Bruce Doak, Petra Fromme, Uwe Weierstall, Michael H Huang, John C.H Spence, Ilme Schlichting, Brenda G Hogue, Haiguang Liu

**Keywords:** DNA methylation, Retina, Organogenesis

*Scientific Data* 4:170048 doi: 10.1038/sdata201748 (2017); Published 11 April 2017; Updated 24 October 2017.

The Data Descriptor incorrectly states the number of normal incidences used to generate the plot in Fig. 4b as 209. This plot was generated from 32 normal incidence cases.

